# Increased leptin and A-FABP levels in relapsing and progressive forms of MS

**DOI:** 10.1186/1471-2377-13-172

**Published:** 2013-11-11

**Authors:** Silvia Messina, David Vargas-Lowy, Alexander Musallam, Brian C Healy, Pia Kivisakk, Roopali Gandhi, Riley Bove, Taha Gholipour, Samia Khoury, Howard L Weiner, Tanuja Chitnis

**Affiliations:** 1 Partners Multiple Sclerosis Center, Brigham and Women’s Hospital, Boston, MA, USA; 2 Department G.F. Ingrassia, section of Neuroscience, Università degli studi di Catania, Catania, Italy; 3 Center for Neurologic Diseases, Brigham and Women’s Hospital, Boston, MA, USA; 4 Biostatistics Center, Massachusetts General Hospital, Boston, MA, USA; 5 Partners Pediatric MS Center, Massachusetts General Hospital, Boston, MA, USA

**Keywords:** 41. multiple sclerosis, 132. Autoimmune disease, 175. Neuroendocrinology

## Abstract

**Background:**

Leptin and adipocyte-fatty acid binding protein (A-FABP) are produced by white adipose tissue and may play a role in chronic inflammation in Multiple Sclerosis (MS). To assess leptin and A-FABP in relapsing and progressive forms of MS.

**Methods:**

Adipokine levels were measured in untreated adult relapsing-remitting MS (RRMS), secondary progressive MS (SPMS), primary progressive MS (PPMS) and Healthy control (HC). Pediatric-onset MS (POMS) and pediatric healthy controls (PHC) were also assessed. Leptin and A-FABP levels were measured in serum by ELISA. Groups were compared using linear mixed-effects model.

**Results:**

Excluding two patients with Body Mass Index (BMI) > 50, a significant difference in leptin level was found between RRMS and HC controlling for age (p = 0.007), SPMS and HC controlling for age alone (p = 0.002), or age and BMI (p = 0.007). A-FABP levels were higher in SPMS than HC (p = 0.007), controlling for age and BMI. Differences in A-FABP levels between POMS and PHC was observed after controlling for age (p = 0.019), but not when BMI was added to the model (p = 0.081).

**Conclusion:**

Leptin and A-FABP levels are highest in SPMS compared to HC, suggesting a role in pathogenesis of this disease subtype. A-FABP levels are increased in POMS patients and may play a role in the early stages of disease.

## Background

Multiple sclerosis (MS) is a T-cell mediated inflammatory disease, involving both innate and adaptive immunity [1]. Obesity has been associated with a chronic inflammatory state, due to the secretion of pro-inflammatory proteins in the blood [2]. Increased body mass index (BMI) at the age of 18 is associated with a two-fold increase in the risk of MS [3]. A potential reason for the impact of obesity on disease is the associated increase in adipokines, a family of molecules with effects on inflammatory and autoimmune diseases [4].

Leptin is involved in the regulation of food-intake, energy expenditure, and inflammation. In addition to white adipose tissue (WAT), leptin is also produced by lymphocytes [5,6]. Leptin levels have been investigated in relapsing remitting (RR) MS and secondary progressive (SP) MS patients with differing results [7]–[9].

Serum adipocyte fatty acid-binding protein (A-FABP) belongs to a family of small cytoplasmic proteins. A-FABP is produced by WAT and monocytes, and its expression is enhanced by toll-like receptor-2 (TLR-2) stimulation [10]. A-FABP is predictive of poor outcome in ischemic stroke, suggesting it may play a role mediating stroke-associated inflammation [11]. To date, no studies have addressed the role of A-FABP in MS.

Leptin is produced by lymphocytes and can promote the activation of monocyte-macrophages while A-FABP is produced by macrophages. Therefore, leptin and A-FABP may respectively affect the adaptive and innate arms of the immune system, potentially playing critical roles in different stages of MS. We therefore chose to investigate these two adipokines in relapsing and progressive MS patients.

The aim of this study is to evaluate the levels of leptin and A-FABP in different categories of MS patients compared to healthy controls (HC). Since the relationship between protein levels and MS may vary according to developmental age, levels in MS patients were compared to HC both in adult and pediatric onset patients. Finally, in order to investigate a potential role of adipokines in clinical relapses, adipokine levels were studied in patients before or during a relapse.

## Methods

### Patients

Five groups of subjects contributed to the three main analyses of our study.

#### Adult-onset MS (AOMS)

The first group of subjects included patients with onset of MS after age 18 from the Comprehensive Longitudinal Investigation of Multiple Sclerosis at the Brigham and Women’s Hospital (CLIMB) [12]. This study has enrolled and followed longitudinally patients since 2000, with annual standardized clinical exams, MRIs and stored blood samples. Patients were included in this study if they met the following diagnostic criteria: clinically definite RRMS or primary progressive MS (PPMS), by the 2005 McDonald criteria [13]. SPMS patients were identified using the following criteria: relapsing onset of disease, EDSS ≥ 3.0 and evidence of sustained disease progression. Subjects included in this study had not been exposed to intravenous steroids or disease modifying treatment (DMT) for at least 30 days prior to the blood sample, and had full anthropometric data (height and weight) recorded within a year of blood sample collection. RRMS patients were not treated for personal choice or because they were undergoing a medication wash-out period.

#### Adult Healthy Controls (AHC)

As a comparison group, a set of healthy controls (HC) enrolled in the Phenogenetic Project at the Brigham and Women’s Hospital was recruited. HC were matched for age and sex, and both a blood sample and anthropometric data were available.

#### Pediatric-onset MS (POMS)

The second group in our study consisted of pediatric-onset MS patients (POMS). These patients were seen at the Partners Pediatric MS Center at the Massachusetts General Hospital (MGH) for Children. Pediatric MS was defined according to the operational definition established by the International Pediatric Multiple Sclerosis Study Group [14]. POMS patients were included if they were untreated by either steroid or DMT for at least 30 days prior to the blood sample collection and had full anthropometric data available.

#### Pediatric Healthy Controls (PHC)

Age and sex matched pediatric healthy controls (PHC) were also enrolled from MGH for Children.

#### Relapsing AOMS

The third group of patients consisted of CLIMB patients who had blood samples collected prior (Pre-relapse group) or during a relapse (Relapse group). A relapse was defined as the occurrence of a new neurological disturbance lasting at least twenty-four hours [15]. Samples were considered prior to a relapse if the blood was drawn within ninety days before a relapse. For these patients, a sample during remission (if available) was also analyzed for comparison. A remission sample was defined by no relapses occurring six months before, or after the collected sample. Seven patients in the pre-relapse group and one in the relapse group were untreated. The demographic and clinical characteristics of all three groups of subjects are provided in Table 1. Clinical data for all MS patients were validated by a member of the study staff (SM). Institutional Review Board approval was granted by the Partners Human Research Committee.

**Table 1:** Demographic characteristics

	**Group 1**				**Group 2**		**Group 3**	
	**Relapsing- remitting MS**	**Primary progressive MS**	**Secondary progressive MS**	**Healthy controls**	**Pediatric onset MS**	**Pediatric healthy controls**	**Pre- relapse patients**	**Relapse patients**
**N**	20	13	12	48	12	10	18	11
**Males N (%)**	3 (15.0)	6 (46.2)	1 (8.3)	14 (29.2)	4 (33.3)	3 (30.0)	3 (16.7)	2 (18.2)
**Age Mean (SD)**	43.0 (8.9)	56.9 (7.7)	53.6 (12.5)	45.1 (13.1)	13.9 (2.3)	11.4 (3.3)	40.7 (11.3)	36.4 (10.9)
**Hispanic N (%)**	0 (0)	0 (0)	0 (0)	0 (0)	1 (8.3)	1 (10.0)	1 (5.6)	0 (0)
**White N (%)**	18 (90.0)	13 (100)	12 (100)	44 (91.7)	10 (83.3)	8 (80.0)	17 (94.4)	9 (81.8)
**BMI Mean (SD)**	27.7 (5.0)	30.1 (5.4)	30.7 (7.9)	26.0 (6.2)	26.9 (9.2)	21.4 (3.7)	26.2 (4.4)	29.5 (6.3)
**EDSS Median (Range)**	2 (0–4.5)	6 (1.5-7)	5.0 (2.5-8.0)		1.75 (0–2)		1.75 (0–6)	2 (0–3.5)
**Disease Duration Mean (SD)**	11.3 (9.3)	14.5 (7.6)	19.9 (9.6)		1.75 (2.1)		8.1 (6.6)	8.2 (5.5)
**Leptin (pg/ml) Mean (SD)**	23,462.3 (27,708.9)	17,924.6 (14,009.8)	40,412.6 (27,951.8)	13,840.5 (18,447.6)	27,357.3 (20,207.4)	11,177.0 (11,484.7)	24,381.6 (20,350.1)	37,587.1 (26,465.4)
**A-FABP (ng/ml) Mean (SD)**	21.2 (14.6)	34.9 (18.9)	38.8 (15.5)	18.4 (18.8)	26.7 (18.0)	14.7 (7.6)	17.5 (10.5)	27.7 (13.1)

Legend: MS = Multiple sclerosis, BMI = Body mass index, EDSS = Expanded disability status scale, A-FABP = adipocyte fatty acid binding protein, SD standard deviation.

### ELISA

We measured leptin in serum by ELISA (human Leptin, R&D system) with an intra-assay coefficient of variant of 3.2% and an inter-assay coefficient of variant of 8.2%. A-FABP was measured in serum by ELISA (human A-FABP, BioVendor) with an intra-assay coefficient of variant of 1.4% and an inter-assay coefficient of variant of 6.4%. For each measure, the experiments were completed in batches of up to 40 patients per batch. Some samples were measured in multiple batches. All the samples were assayed in duplicates.

### Statistical analysis

To assess differences between RRMS, SPMS, PPMS, and HC in the AOMS subjects, log-transformed leptin and log-transformed A-FABP levels were compared using a linear mixed-effects model to account for repeated measures on some of the patients. Log-transformation was used due to the skew of the data. For each outcome, the analysis was completed controlling for batch/age and batch/age/BMI. BMI was calculated as body weight(kg)/height(m [2]). Analyses are reported with and without controlling for BMI because BMI may be either an intermediary or a result of the relationship between MS status and leptin/A-FABP rather than a confounder. Pairwise differences among the groups were calculated and a Bonferroni alpha level of 0.0083 was used to assess statistical significance. The interaction between BMI and MS status in the linear mixed-effects model was assessed. Pearson Correlation was used to assess the relationship between leptin and A-FABP levels in both the AOMS and HC subjects, using last available batch measured level where repeated measures were available. Within the AOMS subjects, leptin/A-FABP levels association with the clinical measures (EDSS and disease duration) was assessed using a linear mixed-effects model to account for the repeated measurements. The differences between POMS and PHCs were estimated using the same models as above. Pearson correlation was used to test the relationships between leptin and A-FABP levels and clinical measurements (EDSS and disease duration) within the POMS subjects. To assess the differences in leptin and A-FABP levels when stable and prior to a relapse/during a relapse, linear mixed effects models with a patient specific effect were fit in both the Pre-relapse and Relapse groups separately. For all analyses, p < 0.05 was considered statistically significant and 0.05 < p < 0.1 was considered a trend towards statistical significance. All statistical analyses were completed in the statistical software R (http://www.r-project.org) with the nlme library [16].

## Results

### Comparisons of AOMS and HC

**Figure 1:**
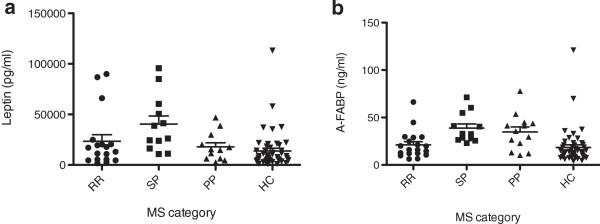
**Leptin and A-FABP levels expressed as mean and SD. a)** leptin levels expressed as mean and SD; **b)** A-FABP levels expressed as mean and SD. A-FABP= adipocyte-fatty acid binding protein, RR=relapsing remitting, SP=secondary progressive, PP=primary progressive, HC=healthy control, MS=multiple sclerosis, SD=standard deviation.

Leptin expression was compared in three groups of AOMS patients (RRMS, PPMS, SPMS) with HC (Table 2 and Figure 1a). In the main analysis controlling for batch/age, a statistically significant difference among the groups was observed (p = 0.008). When the pairwise comparisons were investigated, leptin levels were significantly higher in SPMS compared to HC (p = 0.003). After controlling for batch/age/BMI, the differences between the groups showed a trend towards statistical significance (adjusted p = 0.054), and the SPMS group showed the highest levels of expression. In this model, the association between leptin and BMI was also statistically significant (p < 0.0001).

**Table 2:** Comparisons of log-transformed leptin and log-transformed A-FABP levels in adult onset MS patients to healthy controls

		**Adjusted for batch and age**	**Adjusted for batch, age and BMI**
Leptin	Healthy controls	Reference	Reference
	Relapsing remitting MS	0.66 +/− 0.28 (0.020)	0.43 +/− 0.23 (0.067)
	Secondary progressive MS	1.01 +/− 0.33 (0.003)	0.59 +/− 0.28 (0.041)
	Primary progressive MS	0.16 +/− 0.34 (0.63)	−0.09 +/− 0.28 (0.74)
	Four group comparison p-value	0.008	0.054
A-FABP	Healthy controls	Reference	Reference
	Relapsing remitting MS	0.07 +/− 0.17 (0.69)	0.02 +/− 0.17 (0.90)
	Secondary progressive MS	0.49 +/− 0.21 (0.021)	0.34 +/− 0.21 (0.098)
	Primary progressive MS	0.26 +/− 0.18 (0.16)	0.15 +/− 0.18 (0.41)
	Four group comparison p-value	0.117	0.403

Legend: The estimated difference +/− standard error (p-value) between each group and the healthy controls are presented. MS = multiple sclerosis, A-FABP = adipocyte fatty acid binding protein, BMI = body mass index.

Two patients with BMI > 50 were then excluded from the analysis, given an observed outlier effect in this cohort where all other BMIs were under 40. Subsequently, a statistically significant difference among groups was observed in both analyses (Table 3). Controlling for batch/age, both RRMS and SPMS patients had significantly higher levels of leptin compared to HC (p = 0.007 RR, p = 0.002 SP). Controlling for batch/age/BMI, the difference between SPMS and HC remained significant (p = 0.007). Additionally, leptin levels were significantly higher in SPMS than in PPMS patients (p = 0.0068).

**Table 3:** Comparisons of log-transformed leptin and log-transformed A-FABP levels in adult onset MS patients with BMI < 50 to healthy controls

		**Adjusted for batch and age**	**Adjusted for batch, age and BMI**
Leptin	Healthy controls	Reference	Reference
	Relapsing remitting MS	0.74 +/− 0.27 (0.007)	0.38 +/− 0.23 (0.098)
	Secondary progressive MS	1.07 +/− 0.33 (0.002)	0.76 +/− 0.28 (0.0074)
	Primary progressive MS	0.22 +/− 0.33 (0.49)	−0.14 +/− 0.27 (0.61)
	Four group comparison p-value	0.003	0.014
A-FABP	Healthy controls	Reference	Reference
	Relapsing remitting MS	0.15 +/− 0.16 (0.37)	0.058 +/− 0.16 (0.72)
	Secondary progressive MS	0.63 +/− 0.21 (0.003)	0.54 +/− 0.20 (0.007)
	Primary progressive MS	0.33 +/− 0.17 (0.062)	0.18 +/− 0.17 (0.30)
	Four group comparison p-value	0.020	0.062

Legend: The estimated difference +/− standard error (p-value) between each group and the healthy controls are presented. MS = multiple sclerosis, A-FABP = adipocyte fatty acid binding protein, BMI = body mass index.

When the association between leptin levels in MS subgroups and their BMI was investigated in the entire sample, a trend towards statistical significance (p = 0.065) was observed. The association between BMI and leptin was the lowest in SPMS patients (Additional file 1: Table S1). As with the previous analysis, the extreme BMI patients had a large impact on these results because the BMI by MS subgroup interaction was also not statistically significant when excluding those with BMI > 50 (p = 0.87) (Additional file 1: Table S2).

The A-FABP analysis showed no significant group differences in the four-group (RRMS, SPMS, PPMS, and HC) comparison after adjusting for batch/age and batch/age/BMI (Table 2 and Figure 1b). Excluding high BMI patients, the model adjusting for batch/age showed a statistically significant difference among the groups (Table 3). SPMS patients had the highest levels of A-FABP, and the difference between SPMS and HC was statistically significant (p = 0.003). Adjusting for batch/age/BMI, there was a trend towards a statistically significant effect of group (p = 0.062).

When the interaction between A-FABP in each group and BMI was assessed in the entire sample, a statistically significant difference in the association between BMI and A-FABP based on group was observed (interaction p = 0.019). SPMS patients had significantly lower associations between BMI and A-FABP compared to either HC or RRMS patients (Additional file 1: Table S1). In patients with BMI < 50, the association between BMI and A-FABP levels by MS subgroup interaction was not statistically significant (p = 0.63) (Additional file 1: Table S2). A positive correlation was observed between leptin and A-FABP within both the AOMS (p = < 0.001) and HC (p = 0.003) subjects. However when stratifying the AOMS patients by disease subtype, a significant correlation was observed only in the relapsing subjects (RRMS p = 0.002) (Additional file 1: Table S3). When assessing the relationship between the clinical measures (EDSS and disease duration) and Leptin/A-FABP within the AOMS subjects, we observed a statistically significant association between EDSS and A-FABP (p = 0.024), after adjusting for batch/age. However, after adjusting for batch/age/BMI no significant association was observed in the AOMS samples.

### Comparisons of POMS and PHC

When leptin levels were compared in POMS patients and PHC, no significant differences were observed in either of the analyses (Table 4). The differences between the POMS and PHC were of a similar magnitude as the adult analysis, although the sample size was smaller. No significant interaction between leptin levels, BMI and MS status was observed (p = 0.14).

**Table 4:** Comparisons of log-transformed leptin and log-transformed A-FABP levels in pediatric onset MS patients to pediatric healthy controls

		**Adjusted for batch and age**	**Adjusted for batch, age and BMI**
Leptin	Healthy controls	Reference	Reference
	Pediatric onset MS	1.09 ± 0.73	0.11 ± 0.57
	p-value	0.15	0.86
A-FABP	Pediatric healthy controls	Reference	Reference
	Pediatric onset MS	0.74 ± 0.28	0.49 ± 0.26
	p-value	0.019	0.081

Legend: The estimated difference +/− standard error between each group and the healthy controls are presented. MS = multiple sclerosis, A-FABP = adipocyte fatty acid binding protein, BMI = body mass index.

POMS patients had significantly higher A-FABP levels than controls in the model controlling for batch/age. Controlling for BMI as well, the MS patients had higher levels, but the difference was not statistically significant. No significant interaction between A-FABP, BMI and group was observed (p = 0.77). A significant correlation was observed between the Leptin and A-FABP within the pediatric subjects (p = 0.036). When assessing the relationship between adipokine levels and clinical measures in the POMS subjects, no significant correlations were observed for either leptin or A-FABP.

### Effect of relapse on adipokine levels

To investigate whether adipokine levels change near a relapse in AOMS patients, samples collected within 90 days prior to a relapse (pre-relapse group) or during a relapse (relapse group) were compared to the subject’s own remission samples. Neither leptin nor A-FABP showed significant differences between pre-relapse and remission measurements (estimated increase due to relapse for leptin = 0.081, p = 0.67 and for A-FABP = 0.064, p = 0.44) or between relapse and remission measurements, (estimated increase due to relapse for leptin = 0.36, p = 0.10 and for A-FABP = 0.085, p = 0.40).

## Discussion

Although leptin has been previously studied, this is the first study of A-FABP in MS patients to our knowledge. We found that A-FABP and leptin levels were significantly higher in SPMS patients with a BMI ≤ 50, compared to controls when adjusting for age and BMI. Leptin levels were increased in adult RRMS compared to controls when adjusting for age only, but not when BMI was added to the model. Furthermore, we found a positive correlation between leptin and A-FABP in RRMS patients and POMS, but not in SPMS. Finally, A-FABP levels were higher in POMS patients than in controls when adjusting for age, but not when we additionally adjusted for BMI. There were no differences in leptin and A-FABP levels in patients before or during a relapse compared to the remission phase.

Our analysis performed after excluding the two patients with BMI > 50 is preferred, as the rest of our cohort had a BMI < 40 and these two outliers had high influence on the results. Normal BMI is between 18.5-24.9, and obesity is defined as a BMI of 30 or higher [17]. Mean BMI for U.S. men and women in 2009–2010 is 28.7 [18]. Therefore, these two outliers represent far extremes of obesity well beyond the normal range. It is possible that adipokine levels may be different in extremely obese persons with MS.

BMI could be either a confounder of the effect of MS on adipokines or an intermediary between MS and the adipokines, therefore in our analysis we controlled for age/batch alone and age/batch/BMI in separate models in order to evaluate these relationships. The age/batch model estimates the full group differences, while the age/batch/BMI model estimates the direct effect of group membership on adipokines eliminating any effect of BMI.

Leptin is an adipokine affecting both the innate and adaptive immune system. It acts as an acute-phase reactant and it can be induced by other inflammatory mediators such TNF, IL-1 and IL-6 [19,20]. In the innate system, it promotes the activation of the monocyte-macrophage lineage, activating phagocytosis and secretion of proinflammatory cytokine and leukotriene B [21,22]. Leptin regulates the immune response towards a T helper 1 (Th1) profile [23]. Leptin deficient mice (ob/ob) are resistant to the induction of experimental autoimmune encephalomyelitis (EAE), the animal model of MS [24].

In each model, we found the highest level of leptin in SPMS. The differences between SPMS/RRMS patients and healthy controls decreased controlling for BMI in addition to age and batch. This result shows that some of the differences in leptin expression levels may be due to the increased BMI in RRMS/SPMS compared to controls. Interestingly, the difference between SPMS and HC remained statistically significant even after controlling for BMI, but the difference between RRMS and HC was no longer statistically significant. A potential explanation for these results is that during early stages of disease, leptin levels may be correlated with adipose tissue mass, but with disease progression and age the increased levels of leptin are no longer dependent on adipose tissue and may be produced by other cells including monocytes [10]. Levels of leptin in PPMS were similar to HC, and significantly lower than in SPMS in the model controlling for batch/age/BMI, suggesting that this adipokine is differentially involved in the PP and SP forms of progressive MS, as has been observed for other biomarkers [25]. Leptin levels in SPMS patients have been investigated in a previous study. They found a decrease of leptin and IL-6 levels after IFN-beta 1b 6 months therapy in a group of SPMS patients, suggesting a possible role of leptin as a marker of response to treatment in the progressive stage of the disease [9]. Our samples were gathered in untreated patients (off steroid or DMTs), therefore our findings confirm and strengthen the concept of a possible involvement of leptin in the pathogenesis of SPMS.

A prior study found that leptin levels decrease two months after initiation of IFN- beta1a and increase before a clinical exacerbation in RRMS patients [8]. In a separate study, a higher serum leptin level was observed during remission in RRMS patients than in controls [26]. We did not find a difference in leptin levels in either the relapse or pre-relapse phase compared to remission phases in the same patients, suggesting that acute exacerbations do not cause significant fluctuations in leptin levels.

A-FABP is an adipokine mediating intracellular fatty acid trafficking [27]. It is produced by adipocytes and monocytes after TLR-2 and 4 stimulation [10]. In humans A-FABP levels in the bloodstream are correlated with high obesity index, blood pressure, plasma glucose and reduced High-density lipoprotein cholesterol (HDL) [28]. Indeed a recent study has found a correlation with low-density lipoprotein cholesterol (LDL) and inflammatory MRI measures [29]. A-FABP is associated with stroke in humans and is predictive of a poor outcome [11]. A-FABP, potentiates LPS-induced inflammation, forming a positive feedback with the C-Jun-NH2-terminal kinase (JNK), involved in the A-FABP gene transcription, perpetuating the inflammatory response in macrophages [30]. EAE mice deficient for A-FABP and E-FABP (epidermal-fattyacid-binding-protein) show reduced neurological symptoms and reduced cytokines production, suggesting a possible role of this adipokine in the development of EAE/MS [31].

The highest A-FABP levels were observed in SPMS patients, with similar levels in RRMS patients and HC. In patients with BMI < 50 the difference between SPMS and HC was statistically significant for the model controlling for age/batch, and controlling for BMI as well. WAT and possibly monocyte production of A-FABP may be present in SPMS, and may contribute to the pathogenesis of this disease subtype.

Our POMS patients had higher levels of A-FABP compared to PHC, but this difference was not significant when controlling for BMI, suggesting that BMI influences A-FABP levels. Interestingly, although leptin is involved in the initiation of puberty, [32,33] we did not find a difference in leptin levels between POMS and healthy controls. POMS patients are characterized by a higher relapse rates compared to AOMS patients [15]. Increased BMI at the age of 18 in females and at the age of 20 in males has been related to an increased risk to develop MS, consistent with the hypothesis that the adolescence is a critical window for the development of MS [3,34]. A recent study found increased BMI in female POMS patients compared to demographically matched controls, suggesting that BMI and possibly adipokines may play a role in early-onset disease [35]. During puberty, a state of low-grade inflammation and a consequent increase in adipokine levels may predispose towards autoimmunity.

We found a positive correlation between A-FABP and leptin in RRMS and POMS, suggesting that possibly in the early stage of the disease these adipokines are both involved in the early inflammatory phase of disease, and potentially disease development [36]. It is interesting that this correlation was not observed in the SPMS and PPMS groups, raising the question of independent production of these factors possibly by cells other than adipocytes in later, progressive stages of disease.

Our study has several limitations. First, the relatively small number of patients included, due to the difficulty to obtain untreated patient samples. Second, leptin may exhibit some diurnal variation with lowest levels in the late afternoon [37], however our serum samples were collected throughout the day in all subjects and controls. Third, we could not control for Tanner staging, as these data were not available in the age-matched PHC. While all samples were selected from patients who had been off DMT or intravenous steroids for at least 30 days, it is possible that some DMT despite this washout may affect adipokine levels. Finally in the pre-relapse and relapse group analysis we did not confirm relapses by MRI.

## Conclusions

To the best of our knowledge this is the first study to explore the role of leptin and A-FABP in progressive and relapsing subcategories of untreated adult- and pediatric-onset MS patients. We found the highest levels of A-FABP and leptin in adult SPMS patients, suggesting an intrinsic adipokines dysregulation in this disease subtype. We also found increased A-FABP levels in pediatric MS patients, which may be a marker of early inflammatory disease. Further longitudinal studies are required to study the potential for these adipokines to serve as biomarkers of disease progression, to monitor the effectiveness of therapy. Mechanistic studies addressing adipokine pathway mediators may lead to the identification of novel therapeutics targets. Longitudinal studies investigating the role of adipokines in CIS and POMS patients are needed to elucidate the interaction between BMI, adipokines in the early stages of the disease.

## Competing interests

The authors report no potential conflict of interest for this manuscript.

## Authors’ contributions

SM and DV-L contributed to the design and drafting for intellectual contents of this manuscript. BH and AM contributed to the design, statistical analysis and drafting for intellectual contents of this manuscript. PK, RG, RB, TG, SK and HW contributed to intellectual contents. TC contributed to design and drafting for intellectual contents. All authors read and approved the final manuscript.

## Pre-publication history

The pre-publication history for this paper can be accessed here:

http://www.biomedcentral.com/1471-2377/13/172/prepub

## Supplementary Material

Additional file 1: Table S1 Comparisons of association between BMI and leptin/A-FABP across groups, **Table S2**: Comparisons of association between BMI and leptin/A-FABP across subgroups of MS patients with BMI < = 50, **Table S3**: Correlation between Leptin and A-FABP across subgroups of MS patients (Pearson’s correlation). Click here for file
