# Supplementary material for: Increased leptin and A-FABP levels in relapsing and progressive forms of MS
Source: BMC Neurol. 2013 Nov 11;13:172. doi: 10.1186/1471-2377-13-172 (PMC3829106; doi:10.1186/1471-2377-13-172)
Supplement: Additional file 1: Table S1 — Comparisons of association between BMI and leptin/A-FABP across groups, Table S2: Comparisons of association between BMI and leptin/A-FABP across subgroups of MS patients with BMI < = 50, Table S3: Correlation between Leptin and A-FABP across subgroups of MS patients (Pearson’s correlation). [file 1471-2377-13-172-S1.doc]

**Supplementary Table 1**: Comparisons of association between BMI and leptin/A-FABP across groups

|  |  | Estimated regression coeffieicnt |
| --- | --- | --- |
| Leptin | Healthy controls | 0.11±0.019 |
|  | Relapsing remitting MS | 0.14± 0.041 |
|  | Secondary progressive MS | 0.028± 0.029 |
|  | Primary progressive MS | 0.094± 0.048 |
|  | Interaction p-value | 0.065 |
|  |  |  |
| A-FABP | Healthy controls | 0.049±0.014 |
|  | Relapsing remitting MS | 0.073±0.028 |
|  | Secondary progressive MS | -0.017±0.019 |
|  | Primary progressive MS | 0.035±0.023 |
|  | Interaction p-value | 0.019 |

Legend: The estimated change in log-transformed leptin and log-transformed A-FABP corresponding to a one-unit increase in BMI is presented for each group. MS=multiple sclerosis, A-FABP= adipocyte fatty acid binding protein, BMI=body mass index.

**Supplementary Table 2**: Comparisons of association between BMI and leptin/A-FABP across subgroups of MS patients with BMI<= 50

|  |  | Estimated regression coefficient |
| --- | --- | --- |
| Leptin | Healthy controls | 0.12±0.024 |
|  | Relapsing remitting MS | 0.14± 0.040 |
|  | Secondary progressive MS | 0.089± 0.062 |
|  | Primary progressive MS | 0.094± 0.049 |
|  | Four group comparison p-value | 0.87 |
|  |  |  |
| A-FABP | Healthy controls | 0.033±0.017 |
|  | Relapsing remitting MS | 0.073±0.027 |
|  | Secondary progressive MS | 0.030±0.043 |
|  | Primary progressive MS | 0.036±0.023 |
|  | Four group comparison p-value | 0.63 |

Legend: The estimated change in log-transformed leptin and log-transformed A-FABP corresponding to a one-unit increase in BMI is presented for each group. MS=multiple sclerosis, A-FABP= adipocyte fatty acid binding protein, BMI=body mass index.

**Supplementary Table 3** Correlation between Leptin and A-FABP across subgroups of MS patients (Pearson’s correlation).

|  | *Using last available measurement* | | *Using last available measurement and where BMI is less than or equal to 40* | |
| --- | --- | --- | --- | --- |
| *Patient Group* | *Correlation Coefficient* | *p-Value* | *Correlation Coefficient* | *p-Value* |
| *Healthy Controls* | *0.426* | *0.003* | *0.329* | *0.024* |
| *SPMS* | *0.522* | *0.082* | *0.392* | *0.234* |
| *RRMS* | *0.708* | *0.002* | *0.708* | *0.002* |
| *PPMS* | *0.191* | *0.553* | *0.191* | *0.553* |
| *All MS* | *0.454* | *<0.0001* | *0.418* | *<.0001* |

Legend: MS=multiple sclerosis. A-FABP= adipocyte fatty acid binding protein, BMI=body mass index, SPMS=secondary progressive MS, RRMS=relapsing-remitting MS, PPMS=primary progressive MS.
